# Development of a questionnaire to assess dietary restrictions runners use to mitigate gastrointestinal symptoms

**DOI:** 10.1186/s12970-019-0278-7

**Published:** 2019-02-28

**Authors:** Jill A. Parnell, Hailey Lafave, Kim Wagner–Jones, Robyn F. Madden, Kelly Anne Erdman

**Affiliations:** 1 Department of Health and Physical Education, Mount Royal University, 4825 Mount Royal Gate SW, Calgary, Alberta T3E 6K6 Canada; 2 Department of Biology, Mount Royal University, 4825 Mount Royal Gate SW, Calgary, Alberta T3E 6K6 Canada; 3 Helios Wellness Centres, Teaching, Research, Wellness Building, Suite 402, 3280 Hospital Drive NW, Calgary, Alberta T2N 4Z6 Canada; 4 Faculty of Kinesiology, University of Calgary, 2500 University Drive NW, Calgary, Alberta T2N 1N4 Canada; 5 Sport Medicine Centre, University of Calgary, 2500 University Drive NW, Calgary, Alberta T2N 1N4 Canada

**Keywords:** Exercise-induced gastrointestinal symptoms, Pre-exercise nutrition, Reliability and validity, Endurance running

## Abstract

**Background:**

Exercise induced gastrointestinal (GI) symptoms can plague athletes, especially runners. Sport nutrition recommendations are nutrient rather than foods focused and do not adequately address strategies to reduce GI symptoms. The objective was to develop a valid and reliable questionnaire to evaluate pre-training and pre-racing voluntary food restrictions/choices, reasons for avoiding foods, and gastrointestinal symptoms in endurance runners.

**Methods:**

Validity testing occurred through four Registered Dietitians, three of whom possess Master’s degrees, and a dietetic trainee who provided initial feedback. Additionally, one Registered Dietitian is a Board Certified Specialist in Sports Dietetics (CSSD), and another has an International Olympic Committee Diploma in Sports Nutrition. The second version was sent out to nine different experts who rated each question using a Likert scale and provided additional comments. For reliability testing, the questionnaire was administered to 39 participants in a test re-test format. Kappa statistics and the prevalence-adjusted bias-adjusted kappa (PABAK) were used to assess the reliability.

**Results:**

All questions had an average Likert scale rating of 4/5 or greater. All test re-test results falling under basic information exhibited substantial agreement (kappa ≥0.61). All medical questions including food allergies and intolerances had moderate (kappa ≥0.41) or higher agreement. Responses were less consistent for food avoidances while training (5/28 outcomes) versus racing (0/28 outcomes) with a kappa below 0.41. All reasons for avoiding foods were deemed reliable. Regarding symptoms, side stitch while training and gas while racing were the only flagged categories.

**Conclusions:**

Overall, the questionnaire is a valid and reliable tool to evaluate voluntary dietary restrictions among endurance runners. Future studies can use the questionnaire to assess dietary strategies runners employ to reduce GI distress and optimize performance.

**Electronic supplementary material:**

The online version of this article (10.1186/s12970-019-0278-7) contains supplementary material, which is available to authorized users.

## Background

An athlete’s nutritional preparation prior to exercise plays a key role in optimizing performance, yet the details of this preparation remain largely unstudied. The amount of carbohydrate required pre-exercise has been extensively researched [1, 2]; however, information on the optimal foods to meet these requirements is lacking, as are recommendations regarding the amounts of other macronutrients. Pre-exercise nutrition should consider a multitude of factors including nutrient composition, the potential to promote gastrointestinal issues, and digestibility. Food and fluid intakes during exercise have been studied, however, less is known regarding food intolerances and preferences in the pre-exercise nutrition.

An estimated 30–90% of distance runners experience gastrointestinal (GI) symptoms while running, which has been found, anecdotally, to be an underlying cause of underperformance [3]. Commonly reported symptoms during exercise include flatulence, belching, diarrhea, urge to defecate, epigastric pain, reflux/heartburn, abdominal cramping, nausea, vomiting, and fecal blood loss [3–5]. The underlying factors promoting GI symptoms are believed to result from physiological, mechanical, psychological, and nutritional interactions [3, 5, 6].

GI symptoms are commonly observed in endurance athletes and are affected by the intensity and sporting type [5]. Proposed physiological causes are linked to mechanical irritation and reduction of splanchnic blood flow during exercise [7, 8]. Reduced blood flow can lead to gastrointestinal ischemia resulting in increased permeability, bacterial translocation and inflammation; ultimately presenting as increased GI distress in the athlete [9]. During exercise gastric emptying is slowed and orocaecal transit time increases. Furthermore, there is evidence of nutrient malabsorption, and one or both of these effects may aggravate GI symptoms [10]. Environmental conditions also play a role, as symptoms are increased in warmer (30 °C) as opposed to temperate (22 °C) conditions [11]. Consequently, nutritional strategies are needed to moderate changes in gut physiology occurring with exercise, especially among runners.

Many endurance athletes believe the consumption or avoidance of specific foods and/or fluids prior to exercise can reduce GI distress and optimize performance. For example, it was reported that 41% of non-celiac athletes followed a gluten-free diet at least 50% of the time to reduce GI symptoms during training/competition [12]. The same authors also found that a low fermentable oligosaccharides, disaccharides, monosaccharides and polyols (FODMAP) diet reduced GI symptoms in athletes [13]. Moreover, it has been suggested that foods with high fiber, fat, and fructose can trigger GI symptoms [14, 15]. Despite the importance of nutrition as it relates to exercise induced GI symptoms, the current position paper on Nutrition and Athletic Performance does not make recommendations regarding specific foods/food groups in the pre-exercise period. General guidelines to avoid foods high in fat, fiber, and protein are provided with little specificity. The position paper recommends that athletes determine their own food intolerances and stick to a diet that optimizes performance [2].

An evaluation of the dietary restrictions endurance runners have developed by personal trial and error will provide an entry point in the investigation of foods/food groups that optimize performance while minimizing exercise induced GI symptoms. The objective of the study was to develop a valid and reliable questionnaire to assess pre-training/pre-racing voluntary food restrictions, food choices, reasons for avoiding foods, and GI symptoms in endurance runners. The questionnaire will become a valuable tool for researchers to identify pre-exercise nutritional strategies used by endurance runners.

## Methods

### Questionnaire development

The study investigators, two Registered Dietitians with a Certified Specialist in Sports Dietetics (CSSD) and Master’s degrees, one of which was an Olympic cyclist, and the other a competitive distance runner, and an academic (PhD) with sport nutrition expertise, developed a draft version of the questionnaire that included basic demographics, running experience and events, medical information, food allergies and intolerances, foods avoided/chosen prior to endurance running, GI symptoms experienced, and reasons for avoiding foods before running. Questions regarding sources of nutrition information were also included. The responses were provided by checking boxes or ranking, with the exception of foods chosen, which were open-ended questions. For the content validity testing, the draft version was sent to five experts in the field: four Registered Dietitians with their Master’s degree and one dietetic intern. Additionally, one Registered Dietitian is a CSSD, and another has an International Olympic Committee Diploma in Sports Nutrition. All experts provided written feedback, which was incorporated into the development of the second draft. The second draft was sent out to three different academics with doctorate degrees in nutrition, one Registered Dietitian, and five coaches, all of whom include running in their training programs. Two of the academics have extensive research in sports nutrition and one in the development of nutrition questionnaires. The Registered Dietitian specializes in gastrointestinal disorders. These experts provided written feedback and rated each question using a Likert rating scale with 1 = unacceptable, 3 = acceptable, 5 = highly acceptable. Further amendments were made based on their comments to obtain a final draft. A copy of the questionnaire is available [16] and included as a supplemental file (see Additional file 1).

### Participants

The questionnaire was administered to endurance runners who were 18 years of age or older. It was estimated that thirty-one participants were required for the test re-test based on the null hypothesis of kappa equal to 0.4, true kappa of 0.9, a proportion of positive ratings of 30%, two-tailed significance value of 0.05, and power of 80% [17]. The athletes were recruited from running groups upon approval from the organizers. The Mount Royal University Human Research Ethics Board approved the study (ethics ID 2016–38). All participants provided voluntary, written, informed consent.

### Test re-test protocol

Reliability was determined using the test re-test method. Participants completed the questionnaire twice, with a minimum of one week and a maximum of one month between the initial test and subsequent re-test. The purpose of the test re-test procedure was to investigate the reliability of the questions based on the agreement of participants’ responses.

### Statistical analysis

The kappa statistics, using Cohen’s method, was calculated for all categorical questions [18]. Questions where the participants were asked to rank their top sources of information and preferred sources of information were coded as “yes” or “no” responses for the kappa calculation. Age was evaluated using a Pearson correlation coefficient, as it is a continuous variable. Kappa is the measure of true agreement: it measures the proportion of agreement expected beyond that of chance [19]. The range of possible kappa values is from − 1 to 1, usually falling between 0 and 1. One represents 100% agreement, while 0 represents that agreement is no better than that expected by chance. A negative kappa value indicates that the agreement is worse than that expected by chance [19]. When interpreting kappa values 0.01–0.20 = slight agreement, 0.21–0.40 = fair, 0.41–0.60 = moderate, 0.61–0.80 = substantial, and 0.81–0.99 = almost perfect. The kappa value is determined using the observed agreement and the expected agreement [19]:

$$ \mathrm{K}=\frac{\mathrm{Observed}\ \mathrm{agreement}\hbox{-} \mathrm{Expected}\ \mathrm{agreement}}{1\hbox{-} \mathrm{Expected}\ \mathrm{agreement}} $$

Prevalence and bias play a role in the determination of the kappa value; therefore, kappa can be adjusted to account for high or low prevalence. According to Sim & Wright [17], the adjusted kappa is referred to as PABAK: prevalence-adjusted bias-adjusted kappa and can be calculated as follows [20].

$$ \mathrm{PABAK}=\left(2\ \mathrm{x}\ \mathrm{Observed}\ \mathrm{Proportional}\ \mathrm{Agreement}\right)\hbox{-} 1 $$

The unadjusted kappa and the adjusted kappa (PABAK) values were calculated because the response prevalence for several items was skewed; thus, the unadjusted kappa values were not indicative of the true reliability of the question. For example, the unadjusted kappa values are zero when there is 100% agreement, but only responses from one category (i.e. all no responses for celiac disease); the PABAK adjusts for the low prevalence in one response category and high prevalence in the other response category and presents a value of 1 indicating 100% agreement. We considered the PABAK value when the prevalence index was 0.8 or greater (i.e. when 80% or more of the sample responded in the same direction) or the bias index was greater than 0.15. The same cut-offs for the PABAK assessment were used as for the unadjusted kappa [19]. All statistical tests were conducted using SPSS statistical software version 23 (IBM, Armonk, New York, USA) and STATA S/E version 15 (StataCorp LLC, College Station, TX, USA).

## Results

For the validity testing, all questions had an average Likert scale rating of 4/5 or greater. With respect to reliability, thirty-nine participants (37% male) completed the initial and re-test questionnaire. The questionnaire took approximately 10 min to complete. The mean (SD) age of the group was 45 [14] years. The participants represented a range of performance levels, running experience, and race distances (Table 1). With respect to medical conditions, two reported inflammatory bowel disease (IBD), two reported irritable bowel syndrome (IBS), six reported heart burn, and one reported hiatus hernia.

**Table 1:** Participant characteristics

Characteristics	Participants n (%)
Performance Level	
Recreational lower half of age group	18 (46)
Recreational upper half of age group	15 (39)
National/International	1 (3)
Don’t compete	5 (13)
Run Hours per Week	
0–5 h	19 (49)
5–10 h	15 (39)
10–15 h	4 (10)
20–25 h	1 (3)
Years Running	
0–3 years	6 (15)
3–5 years	7 (18)
5–7 years	4 (10)
7+ years	22 (56)
Running Distance	
5 km	7 (18)
6–10 km	15 (39)
½ marathon – 21 km	7 (18)
Marathon – 42 km	5 (13)
Ultra-marathon	2 (5)
Don’t Compete	3 (8)

Assessment of reliability for all test re-test results falling under demographic and running experience (gender, performance level, running hours per week, years running, and competition distance) exhibited kappa values above 0.61, demonstrating substantial agreement. Age had 100% agreement (r = 1.0). Test/ retest results for medical information are presented in Table 2. All questions had a moderate agreement or greater.

**Table 2:** Test re-test results for medical information

Variable	% Observed Agreement	Kappa	95% CI	PABAK	PI	BI
Food Allergies						
Milk	97.44	.66	.03–1.00	.95	.92	.03
Whey	97.44	.00	.00–1.00	.95	.97	.03
Gluten	97.44	.00	.00–1.00	.95	.97	.03
Casein	97.44	.00	.00–1.00	.95	.97	.03
No allergies	94.87	.86	.66–1.00	.90	.54	.05
Food Intolerances						
Gluten free	97.44	.00	.00–1.00	.95	.97	.03
Legumes	97.44	.79	.38–1.00	.95	.87	.03
Grains	92.31	.54	.09–.98	.85	.82	.08
Starchy vegetable	97.44	.00	.00–1.00	.95	.97	.03
Cold cereal	94.87	.00	.00–1.00	.90	.95	.05
Fish/seafood	97.44	.00	.00–1.00	.95	.97	.03
Hot cereal	97.44	.00	.00–1.00	.95	.97	.03
Nuts	97.44	.00	.00–1.00	.95	.97	.03
Coffee/tea	97.44	.00	.00–1.00	.95	.97	.03
Yogurt	97.44	.79	.38–1.00	.95	.87	.03
Eggs	97.44	.66	.03–1.00	.95	.92	.03
Energy drink	94.87	.48	−.12–1.00	.90	.90	.05
Cheese	97.44	.79	.38–1.00	.95	.87	.03
Milk	87.18	.47	.08–0.87	.74	.72	.03
Sports drink	97.44	.66	.03–1.00	.95	.92	.03
Lactose-free milk	97.44	.00	.00–1.00	.95	.97	.03
Sports bar/gel	97.44	.00	.00–1.00	.95	.97	.03
Vegetables	89.74	.46	.04–.88	.79	.79	.10
No food intolerances	92.31	.84	.67–1.00	.85	.15	.03

*Kappa* unadjusted kappa, *95% CI* 95% confidence interval, *PABAK* prevalence-adjusted bias-adjusted kappa, *PI* prevalence index, *BI* bias index, *IBD* inflammatory bowel disease. Reliability for medical, allergy and food intolerances was determined using a kappa statistic or PABAK. Celiac disease, irritable bowel syndrome (IBS), heart burn/reflux, hiatus hernia, intestinal parasites, and no medical diagnosis had 100% agreement. IBD, who diagnosed your medical condition, who diagnosed your food allergy, allergy test, and blood allergy test all had substantial agreement. Allergies to tree nuts, sesame, soy, sulfites, egg whites, peanuts, fish/seafood, wheat, mustard, eggs, and monosodium glutamate (MSG) had 100% agreement. Intolerance to soy milk, meat, almond milk, poultry, coconut milk, juice, and fruit had 100% agreement

Questions surrounding dietary restrictions, reasons for avoiding foods and symptoms while training are presented in Table 3. When asked about foods that were avoided pre-training there were twelve flagged categories upon initial assessment. Importantly, however, gluten free grains, water, hot cereal, nuts, fruit, almond milk, and coconut milk were all deemed reliable when the PABAK criteria were considered. All reasons for avoiding foods while training had at minimum a moderate agreement. With respect to symptoms experienced while training, only side ache/stitch had poor reliability with a kappa of 0.37.

**Table 3:** Dietary restrictions, reasons for avoiding foods and gastrointestinal symptoms while training

Variable	% Observed Agreement	Kappa	95% CI	PABAK	PI	BI
Foods Avoided						
Gluten free grain/cereal	94.87	−.03	−.09–0.04	.90	.95	.00
Legumes	94.87	.72	.36–1.00	.90	.79	.00
Water	97.44	.00	.00–1.00	.95	.97	.03
**Grains**	**87.18**	**.38**	**−.04–.80**	**.74**	**.77**	**.08**
Meat	82.05	.49	.17–.80	.64	.56	.13
**Sports bar/gel**	**84.62**	**.17**	**−.25–.59**	**.69**	**.79**	**.05**
**Starchy vegetable**	**84.62**	**.16**	**−.26–.59**	**.69**	**.79**	**.00**
Poultry	89.74	.55	.18–.92	.79	.74	.10
Sports drink	84.62	.44	.09–0.78	.69	.69	.15
Cold breakfast cereal	94.87	.48	−.12–1.00	.90	.90	.05
Fish/seafood	84.62	.48	.13–.83	.69	.64	.05
Juice	84.62	.41	.02–.80	.69	.69	.00
Hot cereal	89.74	.28	−.22–.79	.79	.85	.05
Nuts	94.87	−.03	−.09–.04	.90	.95	.00
Fruit/vegetable smoothie	94.87	.72	.37–1.00	.90	.79	.05
Milk products	87.18	.69	.44–.94	.74	.41	.03
Eggs	92.31	.54	.09–.98	.85	.82	.08
Coffee or tea	82.05	.51	.20–.83	.64	.51	.03
Lactose-free milk	94.87	.47	−.15–1.00	.90	.90	.00
Fruit	94.87	.00	.00–1.00	.90	.95	.05
Energy drink	82.05	.49	.17–.80	.64	.56	.13
Almond milk	92.31	−.04	−.11–.04	.85	.92	.03
Vegetables	94.87	.48	−.12–1.00	.90	.90	.05
Chocolate	82.05	.42	.07–.78	.64	.62	.03
Coconut milk	92.31	−.04	−.11–.04	.85	.92	.03
**High fiber foods**	**84.62**	**.33**	**−.07–.72**	**.69**	**.74**	**.10**
**Soy milk**	**84.62**	**.17**	**−.25–.59**	**.69**	**.79**	**.05**
Don’t avoid foods	79.49	.56	.30–.82	.59	.28	.10
Reasons for Avoiding Foods						
Routine	89.74	.44	−.01–.90	.79	.79	.00
Experience	87.18	.75	.55–.95	.74	.05	.13
Personal preference	82.05	.60	.34–.87	.64	.31	.03
Advice	97.44	.79	.38–1.00	.95	.87	.03
Superstition	97.44	.00	.00–1.00	.95	.97	.03
Symptoms Experienced						
Stomach pain/cramps	84.62	.70	.48–.92	.69	.03	.10
Diarrhea	92.31	.75	.49–1.00	.85	.62	.03
Burping/belching	92.31	.53	.06–1.00	.85	.82	.03
Nausea/vomiting	94.87	.64	.18–1.00	.90	.85	.00
**Side ache/stitch**	**79.49**	**.37**	**.02–.72**	**.59**	**.59**	**.00**
Intestinal issues	84.62	.60	.31–.89	.69	.49	.05
Urge to defecate	89.74	.72	.46–.97	.79	.54	.10
Gas	84.62	.53	.20–.86	.69	.59	.00
Reflux/heart burn	84.62	.53	.20–.86	.69	.59	.05
Bloating	79.49	.54	.26–.82	.59	.33	.00
Fullness/heaviness	79.49	.46	.15–.78	.59	.49	.05
No symptoms	92.31	.68	.35–1.00	.85	.72	.03

*Kappa* unadjusted kappa, *95% CI* 95% confidence interval, *PABAK* prevalence-adjusted bias-adjusted kappa, *PI* prevalence index, *BI* bias index. Reliability for questions regarding pre-training was determined using a kappa statistic or PABAK. Symptoms bleeding had 100% agreement. Responses that did not meet the reliability criteria are bolded

Results for dietary restrictions, reasons for avoiding foods, and symptoms experienced while racing are found in Table 4. All foods had substantial reliability, with the exception of chocolate and starchy vegetables, which had moderate reliability. All reasons for avoiding foods pre-racing had a kappa value of 0.67 or greater. Gas was the only symptom experienced while racing that did not meet the moderate threshold with a kappa of 0.37.

**Table 4:** Dietary restrictions while racing reasons for avoiding foods and gastrointestinal symptoms while training

Variable	% Observed Agreement	Kappa	95% CI	PABAK	PI	BI
Foods Avoided						
Gluten free grain/cereal	94.74	.64	.17–1.00	.89	.84	.00
Water	97.37	.66	.03–1.00	.95	.92	.03
Grains	89.47	.65	.34–.96	.79	.63	.05
Meat	86.84	.69	0.44–.94	.74	.39	.03
Sports bar/gel	92.11	.63	.25–1.00	.84	.76	.08
Starchy vegetable	89.47	.54	.15–.93	.79	.74	.05
Poultry	94.74	.84	.63–1.00	.89	.58	.00
Sports drink	94.74	.80	.54–1.00	.89	.68	.05
Cold breakfast cereal	92.11	.62	.23–1.00	.84	.76	.03
Fish/seafood	89.47	.73	.48–.98	.79	.47	.00
Juice	92.11	.75	.49–1.00	.84	.61	.03
Hot cereal	97.37	.79	.38–1.00	.95	.87	.03
Nuts	94.74	.64	.19–1.00	.89	.84	.05
Fruit/vegetable smoothie	94.74	.77	.46–1.00	.89	.74	.00
Milk products	89.47	.78	.58–.98	.79	.26	.11
Eggs	94.74	.80	.54–1.00	.89	.68	.05
Coffee or tea	89.47	.77	.55–.98	.79	.32	.00
Fruit	97.37	.79	.38–1.00	.95	.87	.03
Energy drinks	89.47	.73	.48–.98	.79	.47	.00
Almond milk	94.74	.64	.17–1.00	.89	.84	.00
Vegetables	94.74	.77	.47–1.00	.89	.74	.05
Chocolate	84.21	.53	.19–.86	.68	.58	.00
Coconut milk	97.37	.84	.54–1.00	.95	.82	.03
High fiber foods	92.11	.72	.43–1.00	.84	.66	.03
Soy milk	97.37	.89	.69–1.00	.95	.71	.03
Don’t avoid foods	86.84	.65	.37–.93	.74	.50	.03
Reasons for Avoiding Foods						
Routine	94.59	.72	.36–1.00	.89	.78	.05
Experience	83.78	.67	.44–.91	.68	.08	.00
Personal preference	94.59	.87	.70–1.00	.89	.41	.05
Advice	97.30	.84	.54–1.00	.95	.81	.03
Superstition	94.59	.00	.00–1.00	.89	.95	.05
Symptoms Experienced						
Stomach pain/cramps	86.84	.74	.54–.95	.74	.03	.13
Diarrhea	92.11	.80	.59–1.00	.84	.45	.03
Burping/belching	94.74	.47	−.15–1.00	.89	.89	.00
Nausea/vomiting	89.47	.54	.14–.94	.79	.74	.00
Side ache/stitch	89.47	.75	.51–.98	.79	.42	.05
Intestinal issues	81.58	.51	.19–.83	.63	.50	.03
Urge to defecate	78.95	.47	.18–.77	.58	.47	.16
**Gas**	**81.58**	**.37**	**.02–.72**	**.63**	**.66**	**.13**
Reflux/heart burn	89.47	.71	.45–.97	.79	.53	.05
Bloating	81.58	.59	.32–.86	.63	.34	.08
Fullness/heaviness	92.11	.68	.35–1.00	.84	.71	.03
No symptoms	97.37	.84	.54–1.00	.95	.82	.03

*Kappa* unadjusted kappa, *95% CI* 95% confidence interval, *PABAK* prevalence-adjusted bias-adjusted kappa, *PI* prevalence index, *BI* bias index. Reliability for questions regarding pre-training was determined using a kappa statistic or PABAK. Avoid legumes, avoid lactose-free milk and symptoms bleeding had 100% agreement. Responses that did not meet the reliability criteria are bolded

Questions regarding current sources of information and preferred sources of information asked the participants to rank their top five or top three options respectively. Out of the seventeen sources of information listed, athletes, teammates, and physicians had a kappa below 0.41 indicating poor agreement. When asked if they had attended a workshop on nutrition, the kappa value was 0.83 and their response to the importance of receiving information was kappa 0.62. All preferred means of receiving information had at least moderate agreement with the exception of websites (kappa 0.39).

## Discussion

The pathophysiology of GI distress experienced by endurance athletes is of a heterogeneous nature. Although there are proposed hypotheses, including the mechanical nature of the exercise and physiological changes, the underlying causes remain poorly understood [3]. Clearly, however, nutrition has a key role in minimizing exercise induced GI symptoms. In this context, it is important to explore voluntary pre-exercise food/fluid restrictions endurance athletes are using to mitigate GI symptoms.

The objective of this study was to develop a questionnaire to evaluate food avoidances and choices used by endurance runners to minimize exercise induced GI symptoms and then test it for validity and reliability. The present questionnaire can be deemed valid as it underwent two rounds of content validity testing by a combination of nutrition academics, Registered Dietitians, and coaches. The inclusion of the Likert scale rating allows for quantification of the validity.

Reliability testing was conducted using the test re-test method with 39 participants. Categories with a kappa statistic below moderate agreement (kappa < 0.41) were flagged as having low reliability. According to Lantz and Nebenzahl [21], the relevance of kappa values must take into consideration the issue of prevalence. The symmetrical distribution of agreement, reflected by kappa values, may be skewed in the presence of unbalanced prevalence. For instance, if a research design is investigating a particular trait, yet majority of the population is without this particular trait, it results in the agreement to be largely skewed due to the low prevalence. Although a balanced prevalence nullifies this effect, it is not always possible to incorporate into the research design [21]. As this study spanned a broad range of categories in order to determine specific food/fluid restrictions, the issue of low prevalence was expected. Bias refers to how much the raters disagree on what proportion of the cases are positive or negative. Kappa is higher when there is a large bias than when the bias is small [17]. The adjusted kappa (PABAK: prevalence-adjusted bias-adjusted kappa) was used to control for extreme prevalence and/or bias.

All test re-test results falling under basic information exhibited substantial agreement and were not of concern. Test retest results for medical information found milk intolerance had one of the lowest agreements. There is a common misunderstanding among the general public and even some health care providers, regarding the difference between cow’s milk protein allergy and lactose intolerance [22]. Lactose intolerance is characterized by a deficiency in the lactase enzyme, leaving undigested lactose in the GI tract resulting in distress. Conversely, a cow’s milk protein allergy is characterized by an immunological response when these proteins are ingested. According to Baron [22], many people misinterpret the signs and symptoms of lactose maldigestion as an allergy. Further complicating the issue, not all people with lactase non-persistence will experience intolerance symptoms, there is a dose effect, and symptoms can be related to other digestive disorders [23].

Results for dietary restrictions pre-training exhibited five items with low reliability grains, sports bar/gel, starchy vegetable, high fiber foods, and soy milk. The stem of the question was “When TRAINING are there any foods/fluids that you purposely AVOID in your pre-run MEAL or SNACK (0-4 hours before running TRAINING)? Please check all that apply”. The remaining 23 items had good reliability suggesting the inconsistency is due to the specific food category, not the wording of the question. Interestingly, although the question wording was similar pre-racing “When RACING are there any types of food/fluid that you AVOID in your pre-race MEAL or SNACK (0-4 hours before running RACES/COMPETITIONS)? Please check all that apply” and the food choices were identical, all race options had at least moderate agreement and most had substantial agreement. Studies have shown that pre-performance routines have direct influence on an athlete’s mental and technical performance. Athletes are often consistent with their pre-competition routines in order to optimize performance; however, may be more flexible when it comes to training, given the lower importance [24]. Logically, it is not surprising that the pre-training category exhibited flagged categories as compared to pre-racing. The disagreement may be due to the participants’ tendency to be more lenient and flexible in their pre-exercise nutrition while training as compared to competing.

Poor agreement was observed in training and proportionally lower agreement pre-racing for “avoiding starchy vegetables”, suggesting confusion with respect to this food category. Starchy vegetables (such as potatoes, corn, and peas) have a higher amount of carbohydrates and fiber in comparison to non-starchy vegetables, thereby affecting their digestion. It would be important to consider providing the participants with more examples of starchy vegetables to increase clarity. Given the recent interest in the impact of FODMAP diets on GI distress [13] one could consider categorizing the fruits and vegetables in this respect, however, it is unlikely that the general population would know the FODMAP classification of a food. Grains pre-training also had poor agreement and may reflect confusion regarding a gluten free versus gluten containing grain.

Reasons for avoiding foods pre-training and pre-racing were reliable and these questions will investigate the athletes’ thoughts regarding why they choose to restrict certain foods pre-exercise.

The questionnaire was also designed to assess the symptoms that runners might experience while training or racing should they consume a food that they would typically avoid. Responses were consistent with the exceptions of side ache while training and gas while racing. It is possible that, as some of the symptoms are similar i.e. gas and bloating, the participants are not able to distinguish between these categories, thus creating confusion. A consideration would be to group these categories in the analysis. As with food avoidances, the results were often more consistent with respect to racing versus training. The difference could be due to the intensity of exercise, as it is reported that symptoms increase with increasing exercise intensity [3], suggesting athletes have a heightened awareness while competing. Additionally, if the athletes were more consistent in their pre-racing diet, it would follow suit that their symptoms would be more consistent while racing.

A secondary objective was to assess sources of dietary advice and potential sources of information. The questions asked participants to rank a top number of options from a selection. In general, the reliability of these questions was at least moderate. The questions should, however, be reworded to ask the participants to “check all that apply” rather than rank, given that they were analyzed as “yes” or “no”. Furthermore, this wording aligns with the wording in the other questions.

The questionnaire is limited in that it does not ask the participant to indicate the reason for avoiding each food, simply their overall reasons. Although this information would be of interest, logistically with 27 food options plus the open-ended “other” and five reason options plus the open-ended “other” it would have made the questionnaire too cumbersome. Conversely, the questionnaire assesses all food avoidances and all reasons for avoiding foods; thus, can indicate reasons for avoiding foods in general. The test re-test would also have benefited from a larger sample size, especially for the questions with a low prevalence; however, to be transparent about the precision of reliability estimates based on the small sample size, the 95% confidence intervals and % observed agreement were provided. Finally, the test re-tests typically occurred two weeks apart; therefore, the questionnaire cannot be considered to provide an indication of the reliability of the responses over a longer timeframe and should be viewed as a cross-sectional tool.

## Conclusions

The questionnaire is a valid and reliable tool to assess pre-training and pre-racing nutrition, as it relates to exercise induced GI symptoms. Future research should focus on administering the questionnaire to runners in a fully powered study. Furthermore, the questionnaire can easily be adapted to other endurance sports and demographics. The information gained from administering this questionnaire will provide the foundation for the development of evidence-guided recommendations to optimize performance in endurance runners.

## Additional file

Additional file 1: Food Restriction in Running Questionnaire. (PDF 116 kb)

## Abbreviations

BI: Bias index
CI: Confidence interval
GI: Gastrointestinal
IBD: Inflammatory bowel disease
IBS: Irritable bowel syndrome
PABAK: Prevalence-adjusted bias-adjusted kappa
PI: Prevalence index
